# Safety during outreach activities in eye care

**Published:** 2021-07-20

**Authors:** Ramasamy Meenakshi Sundaram, Esmael Habtamu

**Affiliations:** 1 Senior Manager – Clinical Services: Aravind Eye Hospital, Madurai Tamil Nadu. India.; 2 Chief Executive Director: Eyu-Ethiopia Eye Health Research, Training and Service Centre, Bahirdar, Ethiopia and Assistant Professor: International Centre for Eye Health, London School of Hygiene & Tropical Medicine, London, UK.

**There are many risks to consider when offering eye care services outside of established eye care facilities. Risk assessment and careful planning will help to keep patients, staff members, and community members as safe as possible.**

**Figure:**
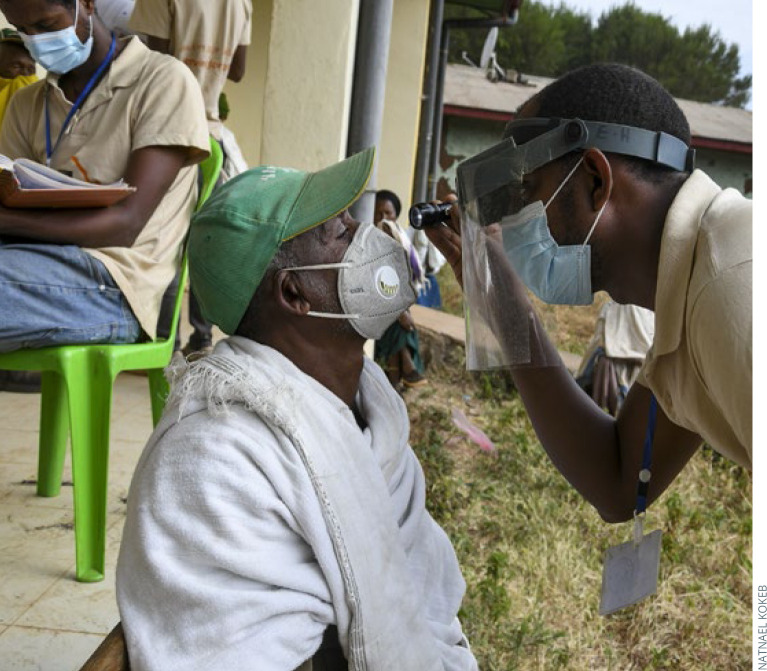
Staff members need personal protective equipment during outreach. **ETHIOPIA**

Outreach services are a popular means of providing eye care in remote and underserved communities. Due to their temporary and infrequent nature, and the need to adapt to changing local circumstances, outreach services may pose a greater risk to staff members and patients than services based at a health facility.

Organisations that are engaged in outreach activities such as mass drug administration (MDA) or surgical camps must carry out detailed assessments to identify risks, and then develop clear protocols and detailed safety guidelines to **mitigate** these risks; i.e., reduce the severity of their impact.

This article highlights what the likely safety issues are during outreach and how they can be addressed.

## Planning

**Figure 1:**
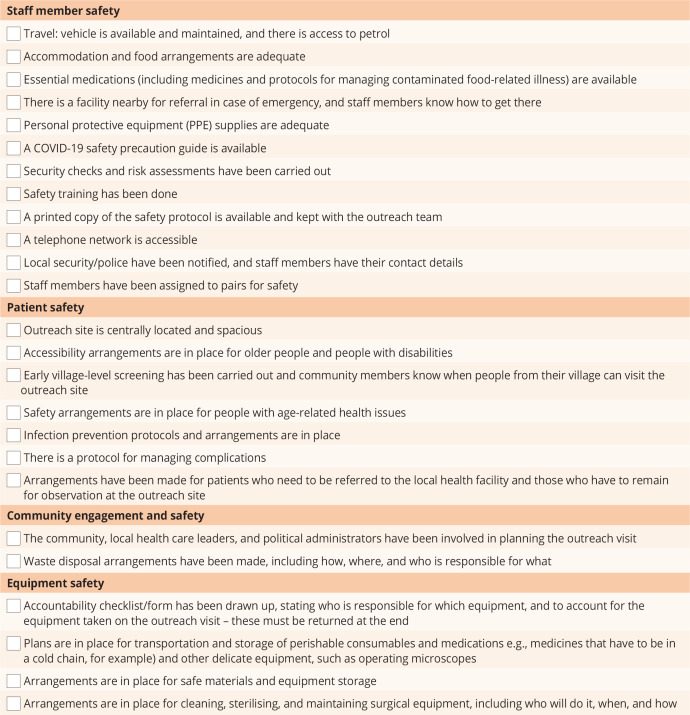
Checklist for safe outreach

Planning an outreach visit must include carrying out a detailed risk assessment and putting together a risk mitigation plan for all the potential safety issues highlighted in the checklist (Figure 1).

The organisation or hospital responsible for the outreach visit must ideally make one person, or a small team, responsible for carrying out the risk assessment, putting together a safety protocol for each activity that will take place during the visit, and providing training to every member of staff involved.

**“A printed copy of the safety protocol should be kept with the outreach team at all times.”**

The outreach team must keep a printed copy of the safety protocol with them at all times, so that it can be referred to whenever the need arises.

## Staff member safety

Staff safety and wellbeing is paramount for successful outreach services.

Basic accommodation and catering facilities – including electricity, water, and toilets – may not be available in remote outreach settings. Planning ahead for access to such facilities by prior arrangement is essential. For example, staff members may have to drive to and from the nearest town where such facilities can be found or pack provisions such as food and water or equipment for sleeping and catering.

Food and water contamination can be a major challenge for staff members during outreach visits. This can be avoided by packing your own food and water. Put together a clear plan for managing food contamination-related illnesses with essential medications such as antibiotics and/or rehydration fluid, or where to refer severely ill staff members if needed. Nearby health facilities that may provide support for such occasions need to be identified in advance.

Adequate supplies of personal protective equipment (PPE) such as gloves, hand sanitisers, disinfectant solutions, and surgical masks and gowns must be available and transported to site. Provide specific guidance relating to infection control and precautions during clinical examination, including donning and doffing PPE, just as would be the case in a static clinical setting. Given the current COVID-19 pandemic, standard operating procedures aligned with national and local guidelines need to be prepared and followed, including protocols for identifying staff members who may have COVID-19 before setting off, and measures to be taken if a staff member is suspected of having COVID-19 during outreach.

Some outreach settings may pose unpredictable security risks. In areas where unrest or violence is a possibility, it may be advisable to delay an outreach visit until stability is restored. If not, the following should be considered at the planning stage:

- Have staff members received the necessary safety training?
- Is a paper copy of the safety protocol available and kept with the outreach team?
- Are there any local authority services (or offices) to oversee safety, e.g., a community policing service?
- Is there access to a telephone or mobile network? If not, how could a possible safety issue be reported to the local authorities?
- How far is the outreach site from the nearest health facility that can provide emergency services?

Depending on the type of outreach, and the setting where it is being conducted, it is recommended that at least two team members work together at all times and that the outreach leader and the local security office/service are kept informed of their whereabouts and travel plans. This is particularly important if the outreach activity involves visiting community members in their homes. Pairing up male and female team members is advisable in areas where it is not safe or culturally acceptable for female team members to move around communities on their own (or in all-female pairs). Another reason to work in male-female pairs is that it will be easier for a male or female chaperone to be available when examining patients.

## Patient safety

Outreach activities should ideally take place in a central location within the community, unless it is more appropriate to carry out home visits or door-to-door delivery of treatment. When selecting a location, it is important to consider the following:

- Who the outreach visit intends to serve
- The available space
- Safety and accessibility for elderly patients and people with disabilities.

A central location should help to reduce the potential safety risks if patients and their families have to travel long distances.

Choosing a location with lots of space indoors will reduce the risk of accidents or falls due to overcrowding. It will also minimise the spread of respiratory infections and make it easier for older people and those with disabilities to move around. In resource-limited settings, schools often have more space and infrastructure than health facilities. If there are multiple classrooms, there can be one activity station (vision assessment, history taking and triaging, refraction, intraocular pressure measurement, final diagnosis & treatment advice, counselling, optical sales, etc.) in each classroom. This will reduce crowding and can make movement safer for patients, staff members, and volunteers.

During outreach, one of the major challenges is crowd management if there has been a good response to community mobilisation. Overcrowding may pose safety risks for older people, women, children, and people with disabilities, and it facilitates the transmission of infections such as COVID-19. Conducting village-level screening programmes a few days before an outreach camp, and allocating different days for each village to come forward, can help to avoid unnecessary overcrowding and the associated health and safety risks.

In cataract surgical camps, some older patients may have hearing, cognitive, or orientation impairments. It is important to remember that some patients are in danger of accidentally harming themselves. A system to identify, prioritise and protect such patients needs to be in place. A detailed process and checklist to ensure that every patient is accounted for and gets the intervention she or he needs, in a safe way, should also be in place.

Outreach facilities are not usually clean enough to safely conduct clinically invasive procedures, as they present an infection risk to patients. If such procedures are planned:

- Allow enough time for cleaning and disinfection of the outreach location before accepting patients
- Provide equipment and facilities for equipment sterilisation
- Develop standard operating procedures for how and when equipment will be sterilised, and who will do it
- Develop a plan of action for managing expected and unexpected life-threatening complications.
- Prepare basic facilities and essential medicines in case patients need to be kept overnight; e.g., if they develop complications that need to be monitored.
- Provide safe transport for patients requiring referral and those with bilateral ocular shields/dressings after, for example, cataract surgery.

## Community engagement and safety

Engaging with the community, and with local health care and political administrators, is an important step when organising a successful outreach visit. Such partnerships are needed in order to:

- Plan the outreach visit alongside the relevant stakeholders
- Address any safety issues
- Advertise the outreach service to the community.

The purpose of the outreach visit must be clearly communicated in order to avoid, or directly address, any prior misinformation about the service. Limited awareness among the communities being reached, and lack of engagement by local authorities, may lead to a lack of trust in the service offered, which will reduce attendance. Local authorities and leaders need to be notified of the service to be delivered, the number and type of professionals involved, their contact numbers, and the dates and location(s) where the outreach team will be working. Community stakeholders, including community volunteers (where they are available), and local officials need to take centre stage in the planning to ensure that dates are selected which do not coincide with local community events or religious festivals.

**“The purpose of the outreach visit must be clearly communicated in order to avoid, or directly address, any prior misinformation about the service.”**

Waste created during outreach activities may pose a major health and environmental safety risk to the community if correct disposal is not planned and carried out. Waste disposal needs to be well thought through and responsibility for correct disposal allocated to a dedicated member of the team.

**Figure:**
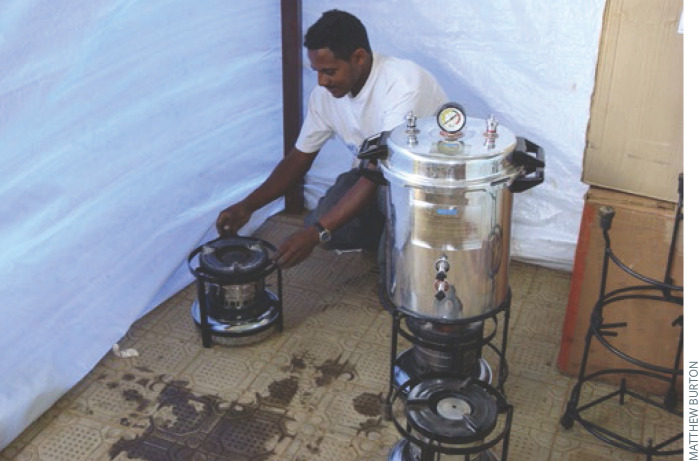
Sterilising surgical equipment during outreach. **ETHIOPIA**
